# Effect of Simvastatin and Atorvastatin on Serum Vitamin D and Bone Mineral Density in Hypercholesterolemic Patients: A Cross-Sectional Study

**DOI:** 10.1155/2014/468397

**Published:** 2014-08-13

**Authors:** K. Abrar Thabit, Abdullah Alhifany, Razan Alsheikh, Sameh Namnqani, Ameen Al-Mohammadi, Soha Elmorsy, Mohammed Qari, Mohammed Ardawi

**Affiliations:** ^1^Clinical Pharmacy Department, Faculty of Pharmacy, King Abdulaziz University, Jeddah 22254, Saudi Arabia; ^2^Clinical Pharmacy Department, Faculty of Pharmacy, Umm Al-Qura University, Makkah 24243, Saudi Arabia; ^3^King Abdullah Medical City, Makkah 24246, Saudi Arabia; ^4^Center of Excellence for Osteoporosis Research, King Abdulaziz University, Jeddah 22254, Saudi Arabia

## Abstract

**Background:**

Besides lipid-lowering effect of statins, they have been shown to have nonlipid lowering effects, such as improving bone health. An improvement in bone mineral density (BMD) has been indicated in some studies after the use of statins, in addition to an increase in 25-hydroxyvitamin D (25OHD) level. The aim of this study is to explore the association between statins and bone health taking into consideration 25OHD level and BMD.

**Methods:**

This is a randomized, cross-sectional comparative study. Subjects were divided into two groups, hypercholesterolemic participants taking simvastatin or atorvastatin as the study group and a matched control group not taking statins. All participants were assessed for serum 25OHD and BMD at lumbar spine and femoral neck.

**Results:**

A total of 114 participants were included in the study, 57 participants in each group. Results of serum 25OHD showed no significant difference between study and control groups (*P* = 0.47), while BMD results of lumbar spine and femoral neck showed significant difference (*P* = 0.05 and 0.03, resp.).

**Conclusion:**

Simvastatin and atorvastatin, at any dose for duration of more than one year, have no additive effect on 25OHD level but have a positive effect on the BMD.

## 1. Introduction

Statins are a group of medications used to treat dyslipidemia. They work by competitively inhibiting HMG-CoA reductase, the enzyme involved in the conversion of HMG-CoA to mevalonate, an early rate-limiting step in the synthesis of cholesterol in the liver [1]. Besides the lipid lowering effect of statins in the primary and secondary prevention of coronary artery diseases [2] and heart failure [3], they have been shown to have nonlipid lowering effects, such as in the treatment of infections [4], Alzheimer's disease [5], stroke [6], and rheumatoid arthritis [7]. Therefore, statins have become an area of research in the battle for management or prevention of many detrimental diseases. The most frequently used statins in Saudi Arabia are simvastatin and atorvastatin. Both medications proved to have a LDL-C lowering effect of >25% and 66%, respectively [8].

Osteoporosis is a leading public health problem and one of the most common diseases among Saudi population as it has a prevalence of 23% [9]. Osteoporosis is defined as the reduction in the strength of bone leading to an increased risk of fractures. Loss of bone tissue is associated with deterioration in skeletal microarchitecture. The World Health Organization operationally defines osteoporosis as a bone density that falls 2.5 standard deviations (SD) below the mean for young healthy adults of the same gender, also referred to as a *T*-score of –2.5 [10].

**Figure 1:**
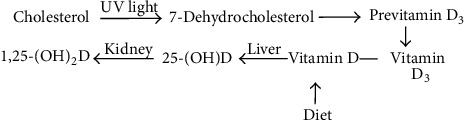
Synthesis and metabolism of vitamin D [14]. 25-(OH)D: 25-hydroxyvitamin D; 1,25-(OH)_2_D: 1,25-hydroxyvitamin D.

Vitamin D is converted to 25-hydroxyvitamin D (25OHD) in the liver. It is a prohormone rather than a true hormone, because it must be further metabolized to its active form, 1,25-dihydroxyvitamin D, in order to gain biologic activity [1]. In Figure 1, 1,25-dihydroxyvitamin D stimulates the intestinal absorption and decreases urinary excretion of calcium and phosphate, the major mineral constituents of bone. It also stimulates bone formation. Factors such as UV exposure [11], race [12], and dietary intake [13] are all known to affect concentrations of 25OHD in humans. Vitamin D deficiency is defined by most experts as a 25OHD level of less than 25 nmol/L. A level of 25OHD of 25 to 75 nmol/L can be considered an indication of a relative insufficiency of vitamin D, and a level greater than 75 nmol/L can be considered sufficient. Vitamin D insufficiency is an important risk factor for osteoporosis and fractures.

There is sufficient evidence for the beneficial use of statins in bone health. Statins have been reported to exempt an unexpected additive effect of stimulating bone formation in animal studies [15]. Moreover, an improvement in bone mineral density (BMD) has been indicated in human participants [16]. The link between 25OHD status in patients taking statins was illustrated by the significant decrease in vitamin D deficiency after the use of atorvastatin was illustrated in the significant decrease in vitamin D deficiency after the use of atorvastatin [17]. On the other hand, another study did not support the general beneficial effect of simvastatin on bone [18]. It is hypothesized that, since cholesterol is a precursor of vitamin D, inhibiting the synthesis of cholesterol will also inhibit the synthesis of vitamin D (Figure 1). Therefore, this study aims to explore the association between statins to bone health. One of the objectives of this study is to assess bone health and statins intake in relation to osteoporosis in hypercholesterolemic patients. Another objective is to evaluate the efficacy of atorvastatin in comparison to simvastatin in relation to bone health. These objectives will be achieved through evaluating statins' effect on 25OHD level and BMD at the lumbar spine and femoral neck.

## 2. Material and Methods

### 2.1. Study Design

**Figure 2:**
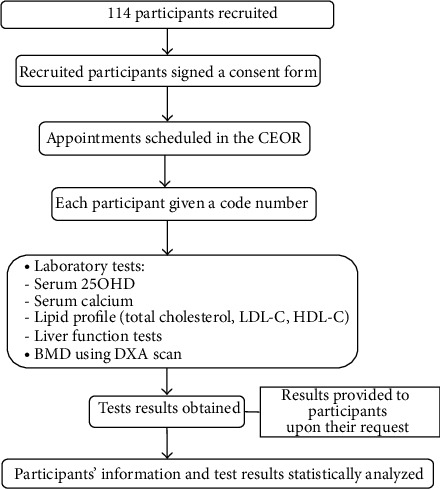
Study process. CEOR: Center of Excellence for Osteoporosis Research; 25OHD: 25-hydroxyvitamin D; LDL-C: Low density lipoprotein cholesterol; HDL-C: High density lipoprotein cholesterol; BMD: Bone mineral density; DXA: Dual-energy X-ray Absorptiometry.

This is a randomized, prospective, cross-sectional comparative study. The study protocol was approved by the IRB of the Center of Excellence for Osteoporosis Research in King Fahd Medical Research Center, Jeddah, Saudi Arabia. Participants were recruited randomly from King Khalid National Guard Hospital, King Abdulaziz University Hospital, and primary healthcare centers. Informed consent was obtained from all participants before enrollment. The study process is illustrated in Figure 2.

### 2.2. Endpoints

The primary endpoint of this study is the effects of statins on 25OHD level, BMD at lumbar spine, and BMD at femoral neck. Secondary endpoints include effects on serum 25OHD and BMD at lumbar spine and femoral neck caused by statin dose variation, variation in their duration of use, and intake of calcium and vitamin D supplementation.

### 2.3. Study Population

Participants eligible for the study were aged 18 years or older, on simvastatin or atorvastatin, in a dose range of 10–60 mg/day of either medication, and as a monotherapy for hypercholesterolemia for duration of one year or longer. We excluded participants on simvastatin or atorvastatin for less than one year, hypercholesterolemic subjects who are on lipid lowering medications in addition to atorvastatin or simvastatin or on statins other than the study medications. Pregnant or breastfeeding women and participants with abnormal liver or renal function tests were also excluded from the study.

Subjects were recruited from King Abdulaziz University Hospital, King Khalid National Guard Hospital, and various primary healthcare clinics (Figure 2). Participants in both groups were matched in terms of age, sex, body mass index, and baseline laboratory values.

Study participants were divided into a study group, hypercholesterolemic participants taking statins, and a control group, participants not taking statins. Participants in the study and control groups were further subdivided based on calcium and vitamin D supplementation. Compliance on statins of study group participants was assessed by personal interviews.

### 2.4. Specimen Collection

Four milliliters of venous blood was withdrawn and collected in coagulated tube under standardized conditions. Serum was prepared by centrifugation at 2500 ×g (1957 rpm) for 10 minutes. Serum and plasma samples were stored. The samples were stored at −85°C within 30 minutes of centrifugation until being analyzed for 25OHD levels and other analytes. All samples were collected between 9:00 and 11:00 am after 12 hours fast.

### 2.5. Bone Mineral Densitometry Measurements

BMD (g/cm^2^) of the anteroposterior lumbar spine (L1–L4) and the means of the proximal right and left femurs (total and subregions) were measured using dual-energy X-ray absorptiometry (DXA) (LUNAR Prodigy Model, Model 8743 BX-1L, Lunar Corp., Madison, WI, USA). A calibration procedure was performed daily to determine instrument variation using a phantom supplied by the manufacturer. Precision error of the phantom was 0.3% and was less than 1.2% for the spine and less than 2.0% for femoral regions* in vivo*. Moreover, there was no significant drift over the period of study using the DXA system. Quality control procedures were carried out in accordance with the manufacturer's recommendations as explained previously [19]. BMD reference values were based on WHO criteria: a *T*-score between −1 and −2.5 indicates osteopenia and a *T*-score equal to or less than −2.5 indicates osteoporosis. A *T*-score of −1 and above is considered normal [10].

### 2.6. Laboratory Measurements

Serum tests were conducted using Vitros 250 Chemistry System Autoanalyzer (Ortho-Clinical Diagnostics—Johnson & Johnson Co., USA). LDL-C level was obtained by calculation from HDL-C level [Total Cholesterol − HDL-C]. Serum 25OHD was measured by direct competitive chemiluminescence immunoassay (CLIA) using a LIASON autoanalyzer (DiaSorin Inc., Stillwater, MN, USA) by inserting 250 *μ*L of serum in the enzyme-linked immunosorbent assay (ELISA). The intra- and interassay CVs were 7.6% and 3.9%, respectively. Other biochemical analytes measured were determined using kits and reagents supplied by Ortho-Clinical Diagnostics, USA, using Vitros 250 Chemistry Autoanalyzer (Ortho-Clinical Diagnostics—Johnson & Johnson Co., USA).

### 2.7. Statistics

The planned sample size of 172 participants with a ratio of control to study 1 : 1 was calculated on the basis of assumption that the study would have a statistical power of 90%. Sample size calculation based on estimated power was done using G∗Power version 3.1 (Heinrich-Heine-University Düsseldorf, Düsseldorf, Germany).* t*-Test (independent 2-samples* t*-test) was used to compare the results of study and control groups, as well as for within-group analyses. One way analysis of variance (ANOVA) testing was used to compare within-group differences in terms of different doses and durations of statins use. Continuous variables are expressed as mean ± SD. Significant two-sided alpha (*P* value) was set at 0.017 as a correction for multiple testing because three outcome variables (serum 25OHD, lumbar spine BMD, and femoral BMD) were studied. A linear regression model was constructed with each of the three variables as dependent variables and study group, statin type and presence of supplementation (calcium and vitamin D) as independent ones. Univariate analyses were run initially then adjusted for supplementation status. All statistics were calculated using SPSS version 17.0 (SPSS, Inc., Chicago, Illinois, USA).

## 3. Results

### 3.1. Study Population

**Figure 3:**
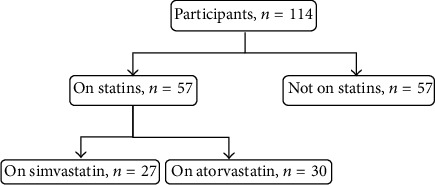
Flowchart of participants in the study.

**Figure 4:**
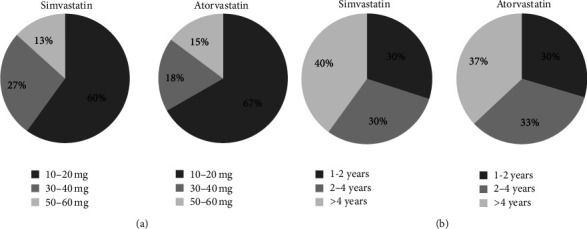
Distribution of study group participants on different doses and durations of use of statins.

**Figure 5:**
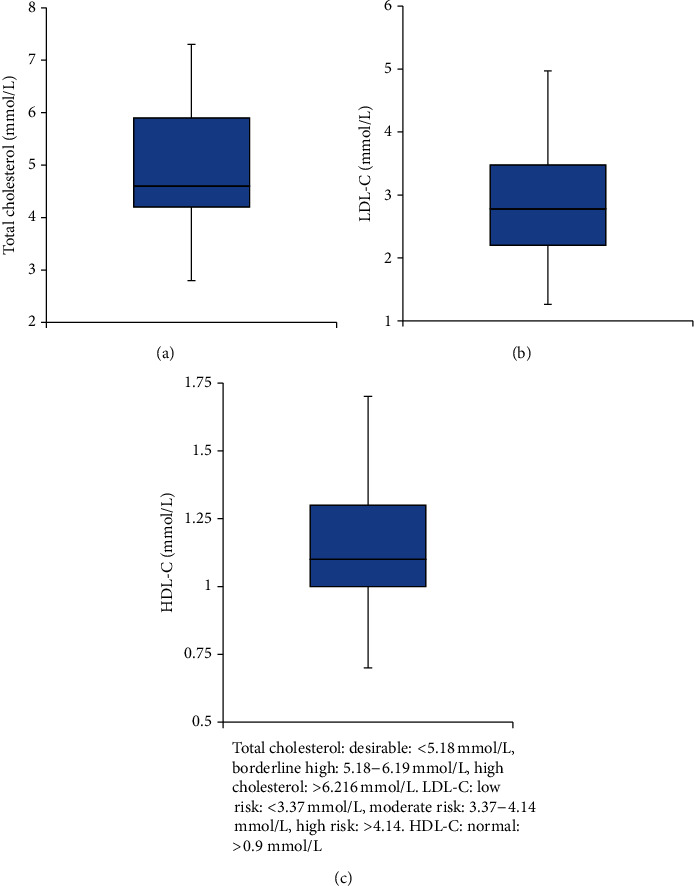
Lipid profile data of the study group. (a) Median and interquartile ranges of total cholesterol; (b) median and interquartile ranges of low-density lipoprotein cholesterol (LDL-C); (c) median and interquartile ranges of high-density lipoprotein cholesterol (HDL-C).

Out of total 165 participants, 51 were excluded from the study because they did not meet the matching criteria. The remaining 114 participants (69%) were included in the study and were divided into a matching of study and control groups with a ratio of 1 : 1. As a result, each group involved 57 subjects.  Figure 3 shows participants disposition in the study. Baseline characteristics of the two groups are presented in Table 1. The age range of the matched participants was 40–75 years. There was no significant difference between the two groups in terms of age, body mass index, liver function, serum calcium, magnesium, and inorganic phosphate. Distribution of study group participants among different doses and durations of statins use was similar between simvastatin and atorvastatin groups (Figure 4). Data from lipid profile tests obtained for the study group had confirmed the compliance of most of the participants on statins which was obtained earlier in the study by personal interviews (Figure 5).

### 3.2. 25-Hydroxyvitamin D Levels

**Figure 6:**
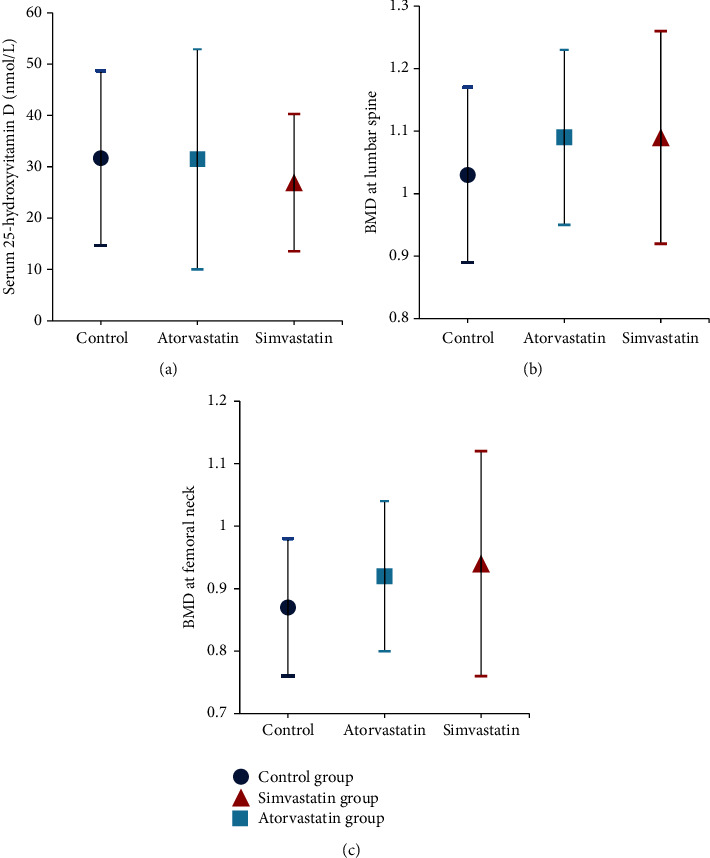
Study group versus control group in relation to serum 25-hydroxyvitamin D and BMD at lumbar spine and femoral neck (mean ± SD). BMD: bone mineral density.

Results of serum 25OHD of the study group showed a mean of 29.32 ± 18.07, while the results of the control group were 31.67 ± 17.03. No statistical difference was found between the two groups (*P* = 0.47) (Table 2) (Figure 6). Relation between statins intake and serum 25OHD was expressed as the regression coefficient.

### 3.3. BMD

Data of lumbar spine BMD revealed a significant difference between the two groups (*P* = 0.05) with a mean of 1.09 ± 0.15 for the study group and 1.03 ± 0.14 for the control group. Similarly, the BMD at the femoral neck also showed a statistical significance between the two groups with *P* of 0.02. The mean of femoral neck BMD of the study group was 0.93 ± 0.15, while that of the control group was 0.87 ± 0.11 (Table 2) (Figure 6). Regression coefficient was used to demonstrate the association of BMD at lumbar spine and femoral neck to the intake of statins.

### 3.4. Subgroup Analyses

When dividing the study group into two subgroups, simvastatin and atorvastatin groups, data showed no significant difference in any of the measured variables, 25OHD, lumbar spine BMD, and femoral neck BMD (*P* = 0.35, 0.92, 0.57, resp.). This was also evident by the insignificant regression coefficient. The mean value of serum 25OHD in the simvastatin group was 26.92 ± 13.36 compared to 31.47 ± 21.45 in the atorvastatin group. The simvastatin group displayed a lumbar spine BMD mean of 1.09 ± 0.17, while the atorvastatin group had a mean of 1.09 ± 0.14. The femoral neck BMD was 0.94 ± 0.18 and 0.92 ± 0.12 for the simvastatin and atorvastatin groups, respectively (Table 3) (Figure 6).

Using ANOVA testing, variation in doses of statins among study group participants did not exhibit an impact on 25OHD level (*P* = 0.23), lumbar spine BMD (*P* = 0.09), and femoral neck BMD (*P* = 0.21). Furthermore, the differences in the duration of statins use were not associated with significant effects on vitamin D levels (*P* = 0.97), lumbar spine BMD (*P* = 0.82), and femoral neck BMD (*P* = 0.94).

**Figure 7:**
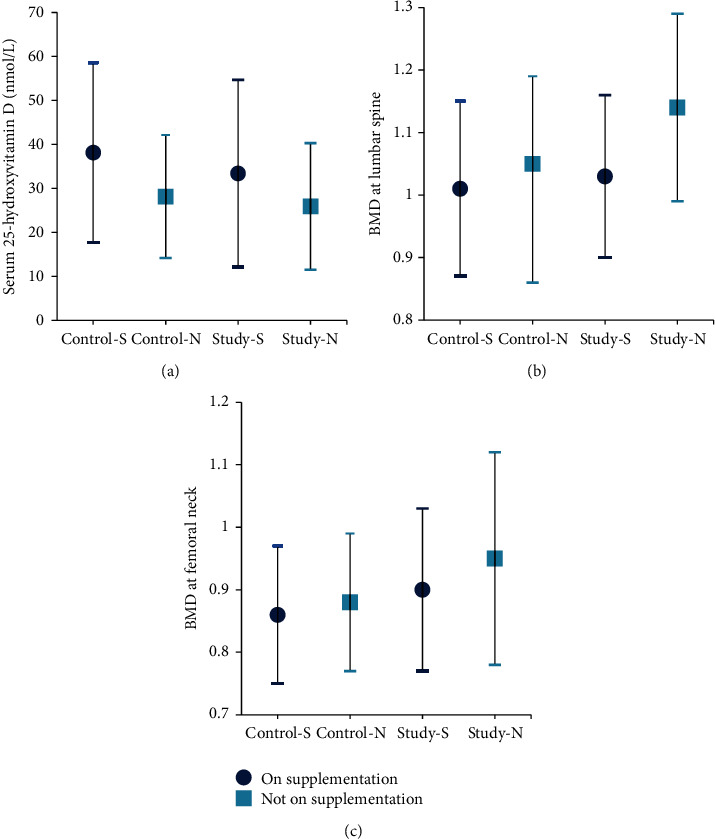
Effect of supplementation on serum 25-hydroxyvitamin D and BMD at lumbar spine and femoral neck (mean ± SD). BMD: bone mineral density; control-S: control group on supplementation; control-N: control group not on supplementation; study-S: study group on supplementation; study-N: study group not on supplementation.

Results of this study demonstrated that 25OHD level may be decreased by using statins. Within the groups analyses showed that participants on supplementation (calcium and vitamin D) in the control group had higher mean 25OHD level (38.14 ± 20.42) than those who were not taking supplements in the same group (28.17 ± 13.98) (*P* = 0.06). In contrast, in the study group, there was no significant difference between the two subgroups with regard to adding calcium and vitamin D supplements (*P* = 0.13). Participants who were taking supplements had a serum 25OHD mean of 33.41 ± 21.24, while those who were not taking supplements showed a mean of 25.89 ± 14.37 (Table 4) (Figure 7).

Interestingly, statins intake along with supplementation had a positive effect on lumbar spine BMD but not on femoral neck BMD. The BMD at the lumbar spine of the study group participants who were taking supplementation showed a mean of 1.03 ± 0.13 versus 1.14 ± 0.15 for those who are not on supplementation (*P* = 0.004). On the other hand, the mean BMD at the femoral neck was 0.9 ± 0.13 for those taking supplements and 0.95 ± 0.17 for those who were not taking supplements (*P* = 0.19) (Table 4) (Figure 7).

Among control group participants, no significant difference was found between subjects on supplementation and those who are not taking supplements in relation to BMD at lumbar spine (*P* = 0.41) and femoral neck (*P* = 0.62). The mean value of lumbar spine BMD for participants on supplementation was 1.01 ± 0.14, whereas the femoral neck BMD displayed a mean of 1.05 ± 0.14. Participants who are not taking supplements had a mean BMD of 0.86 ± 0.11 at the lumbar spine and 0.88 ± 0.11 at the femoral neck (Table 4).

## 4. Discussion

One hundred and fourteen participants of the total 165 were included in this study. Fifty-three participants were excluded, all from the control group, since they could not be matched with participants in the study group based on age and BMI.

Data of this study showed that statin intake has no positive effect on 25OHD status. In fact, cholesterol is the precursor of 7-dehydrocholesterol, which is consequently converted to the active form of vitamin D after the exposure to ultraviolet light (Figure 1). Based on this fact, the inhibition of cholesterol synthesis by statins will lead to further inhibition of vitamin D synthesis. In contrast, this is in odds with the explanation and conclusion by Pérez-Castrillón et al. [17], where he postulated that the increase in 25OHD level by atorvastatin is a result of inhibition of the HMG-CoA reductase enzyme, which may increase levels of 7-dehydrocholesterol and increase the synthesis of 25-hydroxycholecalciferol.

Although 25OHD level was not improved by statin intake, the BMD values of lumbar spine and femoral neck of participants in the statins group in this study were higher than those of the control group, which can support the theory that statins can improve bone health. Possible mechanisms by which statins increase BMD were illustrated by Cruz and Gruber [15], where statins inhibit HMG-CoA reductase and consequently will inhibit the cascade that leads to the activation of osteoclasts, bone resorbing cells. The other possible mechanism is that statins increase the expression of bone morphogenetic protein-2 mRNA. This protein, bone morphogenetic protein-2, is a growth factor that stimulates osteoblasts proliferation, maturation, and thus, formation of new bone. This positive effect of statins on BMD was moderate as Uzzan et al. [20] concluded in their meta-analysis. In addition, this benefit was also confirmed recently [21]; Rejnmark et al. [18] found that the use of statins is associated with improvement in forearm BMD only, but not in the BMD at lumbar spine and femoral neck, which is in contrast to our study result. This finding might be due to the specific type of population included in the study by Renjmark et al. as they specifically included postmenopausal osteopenic women.

This study revealed that there are no significant differences between the two studied statins, simvastatin and atorvastatin, including their different dosages and durations of use, among study group participants in relation to 25OHD level and BMD at lumbar spine and femoral neck.

Given statins' pleiotropic effects, researchers have hypothesized that those effects might be due to statins stimulating the vitamin D endocrine system either by upregulating 1,25-dihydroxyvitamin D or inhibiting its catabolism [22]. In our study we only assessed the association between 25OHD level and statin intake; therefore, such hypothesis could not be rejected. However, a prospective cohort study is warranted to establish a cause-effect relationship. Limitations of this study also included the unmatching between participants regarding their comorbidities and currently used medications other than simvastatin or atorvastatin. Also, liver and kidney functions, diet, and sun exposure were not taken into consideration, which may play important roles in the activation and metabolism of vitamin D. Post hoc analysis showed that this study had a power of 76%.

## 5. Conclusions

Simvastatin and atorvastatin, at any dose for duration of more than one year, have no additive effect on 25OHD level but have a positive effect on the BMD at the lumbar spine and femoral neck.

## Figures and Tables

**Table 1:** Baseline characteristics of study and control groups. ALT: alanine transferase; AST: aspartate transferase; GGT: *γ*-glutamyl transferase.

Variable	Study (*n* = 57)	Control (*n* = 57)	*P* value
Age (years)	55.84 ± 7.2	55.26 ± 6.9	0.66
Sex (male/female)	23/34	18/39	0.33
Body mass index (kg/m^2^)	32.4 ± 6.5	31.1 ± 5.7	0.23
Albumin (g/L)	41.5 ± 3.7	41.6 ± 3.6	0.89
Alkaline phosphatase (U/L)	85.2 ± 31.1	94.7 ± 92.1	0.46
ALT (U/L)	27.3 ± 11.4	29.3 ± 22.9	0.56
AST (U/L)	25.6 ± 8.8	26.5 ± 14.1	0.71
GGT (U/L)	34.8 ± 17	40.5 ± 32.4	0.24
Serum creatinine (*μ*mol/L)	87.1 ± 40.9	74.2 ± 18.1	0.03
Urea (mmol/L)	5.3 ± 2.6	4.6 ± 1.5	0.1
Uric acid (*μ*mol/L)	313.2 ± 89.5	299.3 ± 85.7	0.39
Serum calcium (mmol/L)	2.3 ± 0.13	2.3 ± 0.12	0.51
Magnesium (mmol/L)	0.7 ± 0.09	0.7 ± 0.1	0.64
Inorganic phosphate (mmol/L)	1.2 ± 0.2	1.2 ± 0.1	0.92
On supplementation, calcium, and vitamin D (%)	45.6	35.1	0.07

**Table 2:** Results of serum 25-hydroxyvitamin D, lumbar spine BMD, and femoral neck BMD in the study and control groups. CI: confidence interval; 25OHD: 25-hydroxyvitamin D; BMD: bone mineral density.

Parameter	Study *n* = 57	Control *n* = 57	Regression coefficient (95% CI)	*P* value
Serum 25OHD	29.32 ± 18.07	31.67 ± 17.03	−2.35 (−8.87–4.17)	0.47
Lumbar spine BMD	1.09 ± 0.15	1.03 ± 0.14	0.05 (−0.002–0.11)	0.05
Femoral neck BMD	0.93 ± 0.15	0.87 ± 0.11	0.6 (0.008–0.11)	0.02

**Table 3:** Results of serum 25-hydroxyvitamin D, lumbar spine BMD, and femoral neck BMD in the simvastatin and atorvastatin groups. CI: confidence interval; 25OHD: 25-hydroxyvitamin D; BMD: bone mineral density.

Parameter	Simvastatin *n* = 27	Atorvastatin *n* = 30	Regression coefficient (95% CI)	*P* value
Serum 25OHD	26.92 ± 13.36	31.47 ± 21.45	−4.55 (−14.12–5.07)	0.35
Lumbar spine BMD	1.09 ± 0.17	1.09 ± 0.14	0.004 (−0.8–0.09)	0.92
Femoral neck BMD	0.94 ± 0.18	0.92 ± 0.12	0.02 (−0.06–0.11)	0.57

**Table 4:** Results of serum 25-hydroxyvitamin D, lumbar spine BMD, and femoral neck BMD in the study and control groups with regard to supplementation. CI: confidence interval; 25OHD: 25-hydroxyvitamin D; BMD: bone mineral density. Supplementation is in the form of calcium and vitamin D.

Parameter		On supplementation	Not on supplementation	Regression coefficient (95% CI)	*P* value
Serum 25OHD	Study	33.41 ± 21.24 (*n* = 26)	25.89 ± 14.37 (*n* = 31)	7.52 (−1.99–17.02)	0.13
	Control	38.14 ± 20.42 (*n* = 20)	28.17 ± 13.98 (*n* = 37)		0.06

Lumbar spine BMD	Study	1.03 ± 0.13	1.14 ± 0.15	−0.11 (−0.19–−0.04)	0.005
	Control	1.01 ± 0.14	1.05 ± 0.14		0.41

Femoral neck BMD	Study	0.9 ± 0.13	0.95 ± 0.17	−0.05 (−0.14–0.03)	0.19
	Control	0.86 ± 0.11	0.88 ± 0.11		0.62
